# Do nurses have barriers to quality oral care practice at a generalized hospital care in Asmara, Eritrea? A cross-sectional study

**DOI:** 10.1186/s12903-020-01138-y

**Published:** 2020-05-20

**Authors:** Zewdi Amanuel Dagnew, Isayas Afewerki Abraham, Ghirmay Ghebreigziabher Beraki, Sibyl Mittler, Oliver Okoth Achila, Eyasu H. Tesfamariam

**Affiliations:** 1 Emergency and Critical Care Unit, Department of Nursing, Orotta College of Medicine and Health Sciences, Asmara, Eritrea; 2 Midwifery Unit, Department of Nursing, Orotta College of Medicine and Health Sciences, Asmara, Eritrea; 3 Emergency, Critical Care and Anesthesia Unit, Department of Nursing, Orotta College of Medicine and Health Sciences, Asmara, Eritrea; 4 Clinical Laboratory Sciences Unit, Department of Allied Health, Orotta College of Medicine and Health Sciences, Asmara, Eritrea; 5 Epidemiology and Biostatistics Unit, Department of Statistics, College of Sciences, Eritrean Institute of Technology, MaiNefhi, Eritrea

**Keywords:** Nurses, Oral care, Barriers, Generalized hospital care, Eritrea

## Abstract

**Background:**

Oral care is a fundamental nursing practice that has a great impact on patient well-being and general health during hospitalization. Nurses are responsible for providing oral care in the hospital, however, they usually implement it unsatisfactorily due to inadequate resources, lack of standard protocol, time shortage and ineffective training. The aim of the study was, therefore, to assess nurses’ barriers to quality oral care practice at a generalized hospital. The information obtained will help in highlighting the magnitude of the problem and in the promotion of oral health, prevention and control of oral diseases, reduction of hospital stays and diseases, and in strengthening healthcare systems.

**Methods:**

A cross-sectional design using mixed (quant-qual) method was applied at a generalized hospital. Data for the quantitative study were collected from all (*N* = 73) diploma and associate nurses through face to face interview with a structured questionnaire. On the other hand, in the qualitative part, head nurses (*n* = 6) and staff nurses (*n* = 7) discretely participated in the focus group discussions (FGDs), whereas matron (*n* = 1), assistant matrons (*n* = 2), and supervisor (*n* = 1) in total 4, participated in the key informant interview (KII). The quantitative and qualitative data were analyzed, respectively, using descriptive statistics and thematic framework analysis.

**Results:**

The majority (93.2%) of participants had barriers performing oral care. The barriers mentioned by the participants were; lack of oral care equipment (91.2%), absence of guidelines (73.5%), shortage of staff (67.6%), time constraints (66.2%), inadequate knowledge (54.4%), poor supervision (47.1%), high work load (44.1%), and not being a priority (33.8%). Moreover, through FGD and KII, four main barriers to oral care were identified namely; inadequacy of resources, knowledge gap in oral care practice, nurse related barriers (perception of nurses and initiative of nurses) and gaps in management.

**Conclusions:**

The study concluded that nurses faced barriers at individual, organizational and ministry level that hindered them from performing standard and effective oral care. Therefore, there is a need for further training, motivation, standardized protocol and provision of equipment and supplies to promote oral health of patients.

## Background

Good oral health is a state of being free from oral disease, pain or infection that limits an individual’s ability to eat, speak and socialize. Periodontitis is one of the most common diseases worldwide, which is closely linked with poor oral hygiene and major non-communicable diseases (NCDs). Oral health and oral hygiene should be recognized as essential components of a healthy lifestyle, however, it has been given rare attention [1]. The oral health of hospitalized patients has been found to decline worldwide, with an increase in the amount of dental plaque and gingival infection which occurred within 7–20 days of hospitalization [2].

Nowadays the interplay between oral health and systemic health is well recognized. It is acknowledged that oral health status is important to life quality and plays an important role in overall patient health, even amongst patients with life threatening conditions [3]. Oral care of hospitalized patients has implications in highlighting the magnitude of the problem and in the promotion of oral health, prevention and control of oral diseases, reduction of hospital stays and diseases, and in strengthening healthcare systems [4, 5]. The World Health Organization (WHO) global oral health program has emphasized the importance of increasing the awareness of oral heath worldwide [4].

Effective oral care provides relief, comfort and an infection free mouth to patients who cannot perform this simple activity [6]. Nurses are in the best position to provide this effective oral care as they are at patients’ bedside 24 h a day. Even though skilled and knowledgeable, nurses are extremely important to make appropriate decisions in patient care, they need to have a standard protocol to assess the oral cavity and perform oral care properly. Moreover, the hospital should be equipped with the basic standard equipment for quality oral care [7]. In a study done in South Africa, 81% nurses stated that they would need better supplies to provide oral care and 52.1% indicated that they had no oral care protocol [8].

In addition, it is important for healthcare professionals, particularly nurses, to have regular in-service training on measures and protocols that promote the oral health of patients [7]. However, oral care training has been given less emphasis compared to other nursing care. Importantly, the oral care training does not contain oral health assessment procedures, the effect on systemic health, and its implication on hospitalized patient’s outcomes [9]. Hence, nurses often lack evidence-based awareness to deliver appropriate oral care [6]. In the study done in South Africa, basic nursing training was the only source of oral care training of 36.5% nurses [8]. The majority of the nurses stated that they need to learn more about oral care (88.5%) and would like more information on research proven oral care standards (89.6%) [8]. Moreover, other studies tried to identify barriers to oral care practices like time constraints, lack of adequate staff and materials, heavy workload, poor supervision, poor teamwork, ineffective training and lack of on the job training, lack of standard protocols, uncooperative behavior of patients, and the unpleasant nature of the procedure [10–13].

The effect of poor oral care extends from physiological to psychological well-being of the patient with a subsequent negative impact on treatment plan [14]. Hence, care of the mouth is considered to be one of the most basic nursing activities. The goal of this study is, therefore, to improve the current ritualistic oral care practice in the country. To the best of our knowledge there is no study done to assess the barriers to oral care among nurses in Eritrea. However, this study intensively tried to assess the barriers that hindered nurses from performing up-to standard oral care through interviews and focus group discussions.

## Methods

### Study design and setting

A cross-sectional design using mixed method (quant-qual) was conducted in February 2018 at a generalized hospital. The hospital is situated in Asmara, capital city of Eritrea. The focus of the study area was the adult medical-surgical in-patient department which includes; medical, surgical, intensive care unit (ICU), ear nose and throat (ENT), emergency and recovery wards.

### Study participants

No sample size determination method was applied for the quantitative study because a census survey or complete enumeration was performed. From a total of 79 diploma and associate nurses in the hospital, six participants did not meet the inclusion criteria (Four were absent during data collection and two were non-respondents), hence 73 nurses were enrolled in the study.

In the qualitative study, the expected number of FGD participants were 14, where it comprised of head nurses (*n* = 7) and staff nurses (*n* = 7), however, head nurse from ENT was on leave so there were a total of 13 participants. The staff nurses (*n* = 7) were selected by purposive sampling. There were 4 key informants namely; supervisor (*n* = 1), assistant matrons (*n* = 2) and matron (*n* = 1).

### Data collection tool and techniques

The quantitative and qualitative data were collected separately and independently at different place and time. This study was approved by the Research Ethics Committee of Asmara College of Health Sciences and Eritrean Ministry of Health with a ref. No.: 11/10/17. The study protocol was explained to all nurses who participated in the study and informed consent forms were signed prior to data collection.

### Quantitative data collection tool and technique

A close-ended questionnaire (see Additional file 1) was prepared and used to collect barriers of oral care practice among the nurses. The questionnaire was adopted and modified by the researchers to suit the hospital setting after reviewing similar studies [11, 12]. The list of barriers included in the question were lack of oral care equipment, absence of guidelines, shortage of nurses, time constraints, inadequate knowledge, poor supervision, high work load, not being a priority, no on the job training, and not enthusiastic. The participants were face to face interviewed in their respective wards which took an average of 10 min.

### Qualitative data collection tool and techniques

Guiding questions for the FGD and Key Informant Interview (KII) (see Additional file 2) were prepared to grasp the ideas of the participants. For instance some of the questions were; “How do you see oral care practice in the hospital? Is oral care given routinely in your respective ward?” and so on. FGD and KII were conducted and transcribed in the main language of the participants, namely, Tigrigna. The FGD was moderated by a senior nurse researcher and assisted by a note-taker, and was audio recorded. The group of researchers involved in transcribing comprised of experienced nurses, bi-linguist of Tigrigna and English, bio-statistician and epidemiologist, and dental therapist. Later on the responses from FGD and KII were collected and jointly translated and transcribed into English by a bi-linguist of Tigrigna and English. We have also back translated the transcriptions into Tigrigna to determine whether the meaning had been changed or not, and no considerable change in the meaning was found. Both of the FGD’s were conducted in the hospital conference hall, while KII was conducted in their offices. FGD’s and KII were performed at different times. The average time spent in FGD and KII was 90 and 60 min, respectively.

### Data analysis

The quantitative and qualitative data analysis were done separately.

#### Quantitative data analysis

Descriptive statistics using SPSS (version 22) was used to compute the frequency and percentage for barriers of oral care practice mentioned by all nurses (*N* = 73). The demographic characteristics and barriers of oral care practice results were presented using tables and bar graphs, respectively.

#### Qualitative data analysis

**Fig. 1:**
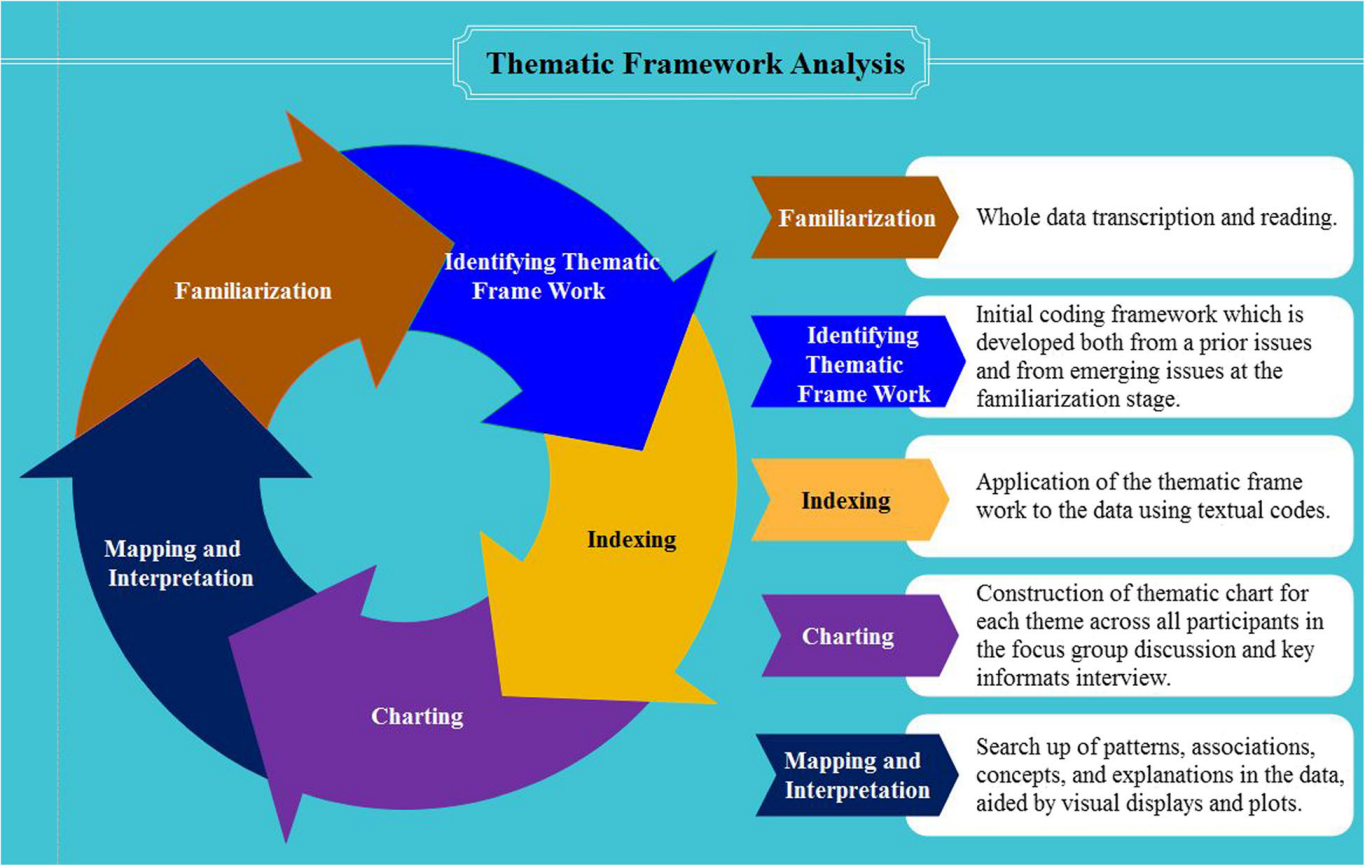
Thematic framework analysis

Analysis was made following the five steps of thematic framework approach, namely; familiarization, identifying thematic framework, indexing, charting, and mapping and interpretation [15] (Fig. 1). Both FGDs and KII were conducted in Tigrigna. Both sets of data were audio-recorded and transcribed. The data were subsequently processed for analysis as indicated above. The transcripts were used to create codes after reading repeatedly. Then, sub-themes were constructed from the codes based on their similarity of contents. The sub-themes were condensed into meaningful themes, in which qualitative description was made. Finally, patterns and association of the constructed themes were identified.

Triangulation of the results from the quantitative and qualitative data were performed.

## Results

### Quantitative data results

#### Demographic characteristics

The median age of the 73 study participants was 26 years, in which majority (46%) of them were between the ages of 20 to 25. More than three quarters (75.3%) of the participants were females. More than half (56.2%) of the participants were diploma nurses and the rest (43.85%) were associate nurses. The median work experience of the participants was 4 years. The participants were working in medical (27.4%), surgical (19.2%), emergency (19.2%), ICU (17.8%), recovery (11%) and ENT (5.5%) wards (Table 1).

**Table 1:** Demographic characteristics of diploma and associate nurses (*N* = 73)

Characteristics	Frequency	Percent
Age, Years, (Median = 26, IQR = 5, Min. = 21, Max. = 54)		
20 to 25	34	46.6
26 to 30	29	39.7
Greater than 30	10	13.7
Sex		
Male	18	24.7
Female	55	75.3
Level of Education		
Associate nurse	32	43.8
Diploma nurse	41	56.2
Experience, Years, (Median = 4, IQR = 5, Min. = 1, Max. = 19)		
1 to 2	25	34.2
3 to 7	34	46.6
Greater than 7	14	19.2
Ward currently working		
Medical	20	27.4
Surgical	14	19.2
Emergency	14	19.2
ICU	13	17.8
Recovery	8	11.0
ENT	4	5.5

#### Barriers to oral care practice

**Fig. 2:**
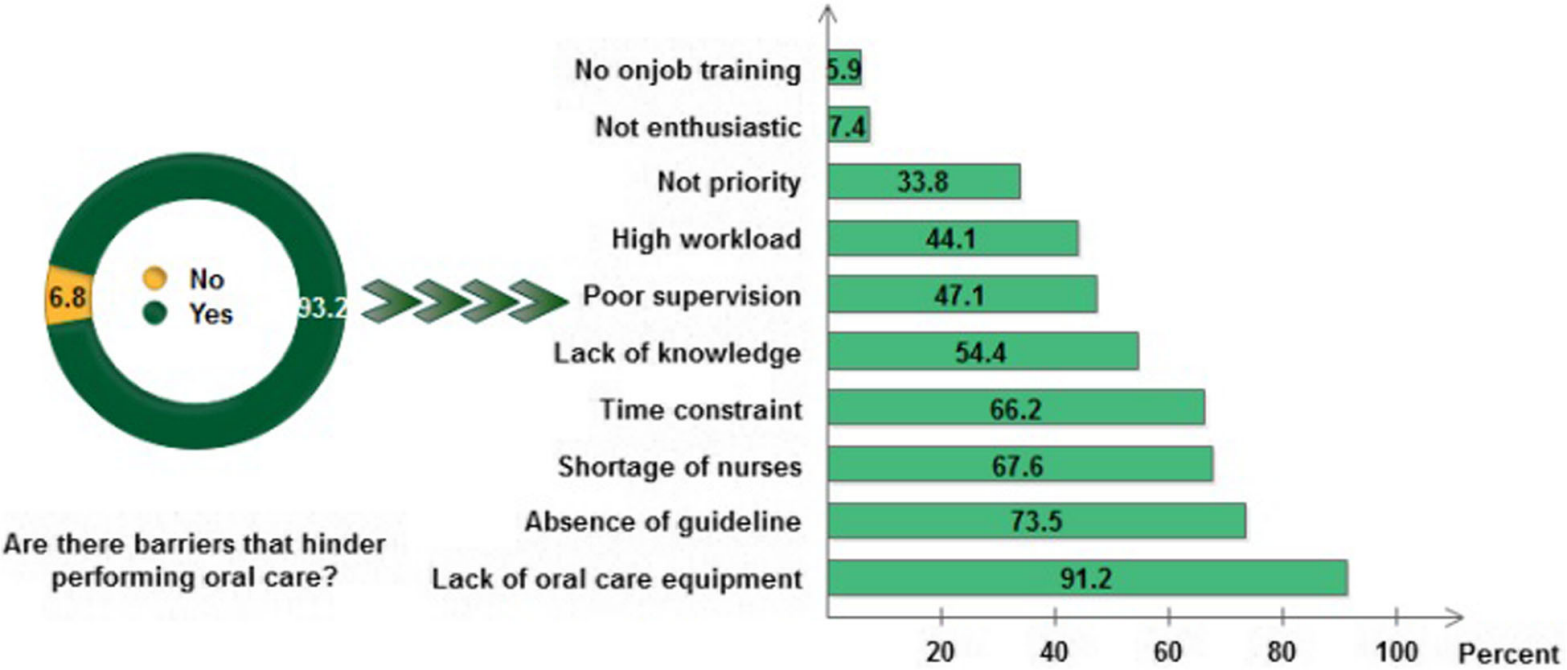
Barriers to oral care among the nurses

The majority (93.2%) of participants had limitations performing oral care. The barriers mentioned by the participants were; lack of oral care equipment (91.2%), absence of guidelines (73.5%), shortage of staff (67.6%), time constraints (66.2%), inadequate knowledge (54.4%), poor supervision (47.1%), high work load (44.1%), and not being a priority (33.8%) (Fig. 2).

### Qualitative data results

#### Demographic characteristics

A total of 17 participants were included in the qualitative study in which 6 head nurses and 7 staff nurses in the FGDs while 4 participants in the KII. Out of the total participants, 76.5% were females. The 20 to 30 and ≥ 51 years age bands accounted for 35.3% of the participants. Moreover, participants in the age of 31–40 and 41–50 years old accounted for 17.6 and 11.8%, respectively. The proportion of their overall working experience in those ≤10 years, ≥ 31 years, 21–30 years and 11–20 years were 52.9, 23.5, 17.6 and 5.9%, respectively. Qualifications’ of FGD and KII participants were diploma nurses (47.1%), associate nurses (35.3%) and degree nurses (17.6%). Moreover, their current position was staff nurses (41.2%), head nurses (35.3%), assistant matrons (11.8%), matron and supervisor each (5.9%) (Table 2).

**Table 2:** Demographic characteristics of focus group participants and key informants (*n* = 17)

Variable	Number	Percent
Sex		
Male	4	23.5
Female	13	76.5
Age (Mean = 41.5, SD = 14.6)		
20 to 30	6	35.3
31 to 40	3	17.6
41 to 50	2	11.8
51 or greater	6	35.3
Qualification		
Degree nurse	3	17.6
Diploma nurse	8	47.1
Associate Nurse	6	35.3
Overall experience (Median = 10.0, IQR = 22)		
10 or less	9	52.9
11 to 20	1	5.9
21 to 30	3	17.6
31 or greater	4	23.5
Position		
Matron	1	5.9
Assistant matron	2	11.8
Supervisor	1	5.9
Head nurse	6	35.3
Staff nurse	7	41.2

#### Identification of themes and sub-themes

**Fig. 3:**
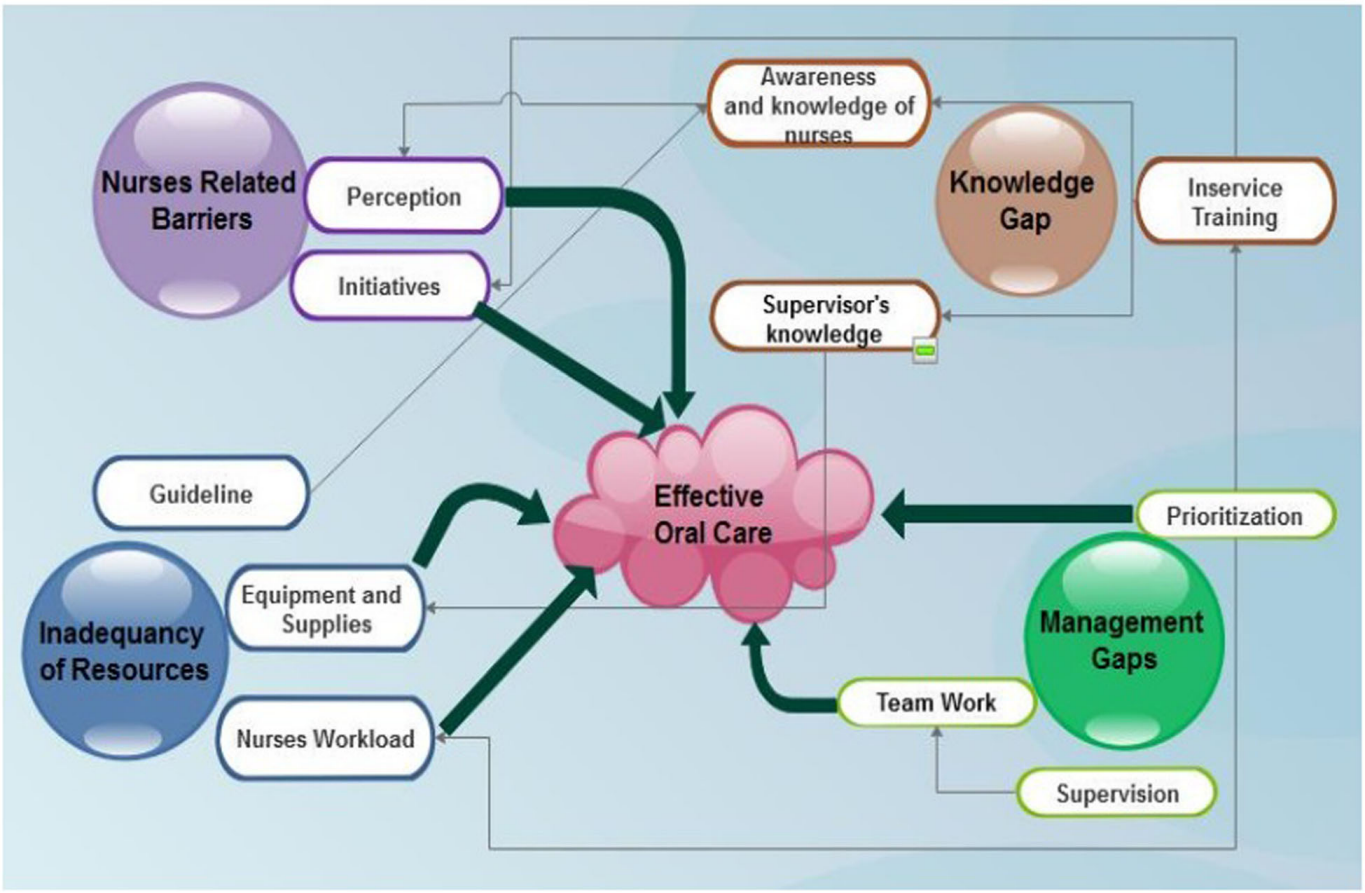
The patterns and interrelationship between themes and sub themes

Seven probing questions guided the conversation throughout the FGD’s and KII in the study. However, the responses were condensed to identify the barriers on quality oral care practice. The four main themes constructed were inadequacy of resources, knowledge gap in oral care practice, nurse related barriers, and management gaps in the hospital. During the familiarization and indexing stages of the thematic framework analysis, the sub-themes were constructed as shown in Table 3. Finally the interrelationship between the themes and sub-themes on effective oral care are displayed in Fig. 3.

**Table 3:** Themes and sub-themes developed for investigating the barriers to oral care practice

Themes	Sub themes
Inadequacy of resources	Guideline
	Equipment and Supply
	Nurses work load
Knowledge gap in oral care practice	Awareness and knowledge of the nurses in oral care
	Knowledge of supervisors in oral care
	In-service training/update
Nurse’s related barriers	Perception of nurses
	Initiative of nurses
Management gaps	Absence of oral care in nursing care plan
	Lack of team work
	Poor supervision

### Inadequacy of resources

#### Guideline

Almost all the staff nurse FGD participants mentioned that there is no oral care guideline in their working station. Whereas some of the staff nurses particularly those who are from medical and ICU wards stated that they were not aware of the guideline concerning oral care in their corresponding wards.

*“I have not seen the guideline regarding oral care. I do not know if it is available or not. Besides, most of us are not interested to ask for the guideline because we do not have a reading habit.”*

On the other hand, the head nurses and key informants noted that the guideline is available in all wards, however, it is locked away, and is thus not available. Although the guideline was provided to the hospital by the nursing division of Ministry of Health (MOH), it has not been updated since 2002.

Another participant reported;

*“There should be sufficient, available and accessible guideline of oral care in the hospital.”*

#### Equipment and supplies

Shortage of equipment and supplies was found to be one of the main barriers. Medical and surgical wards staff as well as head nurses stated that they have an oral care kit. However, the oral care kit was not well equipped, it consists of kidney dish, forceps, and gauze/cotton balls. Besides, there were insufficient oral care kits that can be used for all patients. On the contrary, the other ward head nurses and staff reported not having an oral care kit. They provide oral care using a basin, gauze, and syringe with clean water or normal saline. Sometimes they also use lemon to clean the oral cavity or ask the patients family to bring tooth brush and tooth paste. Participants’ experiences are suggestive of this issue.

*“We have shortage of equipment and supplies like solutions which are especially made for oral care. Previously ERA (Eritrean Relief Association) and the nurses of St. Joseph were helping us with some oral care equipment. Nevertheless, I have observed that there is lack of sincerity in handling equipment by the staff. Once, I asked the MOH for oral care equipment particularly for ICU like solutions, tooth brush and tooth paste, they said we do not have. The MOH does not provide us with adequate oral care equipment and supplies. It is not that expensive but attention is not given to it.”*

Another participant noted;

*“We did not ask for oral care equipment because we thought the oral care kit we have was enough. We did not have clear image of the implication of not practicing an oral care, otherwise, we would have done whatever we can to solve the problem. At least we would have asked patients family to bring tooth brush and tooth paste.”*

#### Nurses work load

According to the statement of the participants, the overall staff allocation in the hospital was insufficient. All focus group participants and key informants stated that there is shortage of staff in all the wards. According to the participants’ estimation, the average nurse to patient ratio was 1:17 during the study period. Oral care has not been a routine care in the wards, even sometimes it was not provided to critically ill patients due to lack of staff and work load. Moreover, nurses give more priority to other nursing activities like medication administration, bathing and vital sign measurement. This shows that oral care is not given priority and seen as a vital care for a patient.

*“Shortage of staff nurses is a big obstacle for us. For instance in the emergency ward there are only 2 or 3 staff per shift and in medical ward there are 35 patients which are being taken care by 2 staff. Hence, the nurses do not give oral care priority in their care.”*

### Knowledge gap in oral care practice

#### Awareness and knowledge of the nurses

Deficits in nurse’s knowledge about standard oral care has been found to be one of the barriers that hindered nurses to perform oral care. Oral care is not merely care which influences the oral cavity, it also determines the general health of the patient. Poor oral care due to lack of adequate knowledge can affect every system of a patient and also results in increased length of hospital stay and hospital bills. However, our focus group participants had inadequate knowledge regarding the possible outcomes of poor oral hygiene. Oral care has been part of the basic nursing practice during education. However, they have not got any updated information regarding oral care procedure, importance and its complications afterwards. Therefore, lack of adequate knowledge and awareness was a major issue raised by the focus group participants. According to the report of the participants, 19.2, 46.6, and 34.2% of the nurses did not take any training for more than 7 years, 3 to 7 years, and 1 to 2 years, respectively.

*“Oral care practice has been ignored. We do only suctioning for the purpose of clearing the air way. We have learned oral care as part of nursing training, however, we have only minimal knowledge about oral care. We perform oral care from experience not depending on scientific ground.”*

Even if oral care was done to critically ill patients it was not up to the standard, because of inadequate knowledge.

*“There is lack of knowledge about oral care. I have not read anything related to oral care ever since I finish nursing training. I have lost everything I had learned about oral care.”*

#### Knowledge of supervisors in oral care

Another factor mentioned by the participants which was presented as a barrier to oral care was inadequate knowledge of the supervisors. Inadequate knowledge of supervisors towards oral care can cause poor supervision of the nurses’ performance. This could be due to the fact that the matron, assistant matrons, and supervisors did not take any training for more than 25 years.

*“Supervisor’s knowledge regarding oral care has never been updated. For a strong supervision of the staff nurses, supervisors need to be knowledgeable.”*

#### In-service training

Training, both pre-service education and in-service training, is one of the most essential mechanisms that promote and update nurses practice of oral care. All focus group participants and key informants stated that there has not been any special training given related to current oral care practice. They have been practicing according to the culture which was adapted in the hospital.

*“There is low awareness related to oral care. However, we have never asked about in-service training as-for oral care because it has not been taken as a major concern. Oral care has been side-lined. It has never been raised as a topic.”*

In general, there was almost no on the job training in the hospital. On the job training is usually given to staff from peripheral health facilities. So, whenever new staff are appointed to the hospital, they get accustomed to the pre-existing practice in the hospital rather than the standard.

### Nurse related barriers

#### Perception of nurses

Generally, oral care is not considered as an essential care for patients in the hospital. Subsequently, it was not done routinely. Even if it was performed, the practice was unsatisfactory (it was given once a day using gauze and normal saline or clean water).

#### Initiative of nurses

Oral care was not given routinely due to many factors, among them was poor initiation and interest by the staff nurses as well as the managers. They lack internal drive to provide nursing care within their scope.

*“Lack of dedication of the staff nurses also does not allow for a routine oral care practices. Nowadays nurses do not have initiative, they work only when they are ordered to do so*.*”*

Another participant stated;

“*Oral care is pure nursing care, nurses should not wait for the order of their superiors. A nurse can do activities which are not harmful to a patient without order. This is the oath of a nurse.*”

### Management gap

#### Poor supervision

All focus group participants claimed that there is low supervision by their superiors. The managers of the hospital do not know if oral care has been done for a patient. Majority of the ward managers do not give attention to oral care. Oral care has been long forgotten topic. Lack of good supervision can reduce nurses work zeal.

“*There is no supervision by our heads or supervisors.”*

Another participant also stated;

*“At the moment, because of the change in the hierarchy of administration, the chain of command is extended. Previously the assistant matron had direct communication with the head nurses but now there is a supervisor in between. So this created a communication problem among assistant matrons and the head nurses. This can affect the supervision negatively. Besides, there seem to exist a problem in decision making by the matron.”*

#### Team work

Lack of team work was also one of the management gaps identified by the key informants.

*“There should be team work among ourselves. Seriousness should be in the work place. Previously, until 2008, we were making discussions among nurses about procedures including oral care. However, now, because of shortage of staff nurse’s, poor initiation, and change in chain of command, we could not do discussion. If there is team work, the insufficiencies in the individual nurse, in the hospital as well as in the MOH can be identified and addressed for a solution. There is no, good collaboration with the dental clinic in the hospital as per the patients’ oral care needs.”*

#### Absence of oral care plan

Since oral care had been ignored and almost forgotten, it has not been part of the daily nursing care plan. For instance medication administration, vital sign measurement, bathing, and bed making are among the daily routine nursing activities. Though oral care is part of this daily care, it was put-aside since its significance was not understood well. It has not been given priority in the nursing care.

*“Oral care should be introduced as routine care in the nursing care plan, and it should be ordered as a nursing care plan.”*

Another participant also noted;

*“According to the standard a nurse should give oral care to patients with self-care deficit. Particularly to ICU patients it is important as they are nurse dependent and hence need special care. However, oral care is done rarely and inadequately*.”

## Discussion

Oral care is a fundamental activity yet very important for the overall health and wellbeing of an individual [13]. Hence, our study assessed barriers to oral care from the perspectives of hospital staff nurses and health care administrators. Overall, our study identified lack of resources, knowledge gaps, weak perception and initiation of nurses, and management difficulties as barriers to proper oral care practice.

Effective oral care signifies an important professional activity in endorsing health and quality of life to patients who are critically ill and cannot perform this simple activity [6, 7]. Nurses are in the best position to provide this effective oral care as they are at patient’s bedside 24 h a day. Skilled and knowledgeable nurses are extremely important, however, the hospital also need to have adequate staff, enough supply of equipment, protocol/guideline, regular in-service training, and constructive supervision to assess the oral cavity, perform oral care properly and prevent poor outcomes of patients [7].

In the current study, oral care was found to be done rarely and inadequately. Hence the study tried to assess the barriers as to why oral care was not a routine care. The quantitative study discovered many barriers addressed by the participants. The main barrier was lack of oral care equipment and supplies followed by absence of a guideline, shortage of staff nurses, inadequate knowledge and poor supervision. This might be attributed to the fact that the hospital is a public sector hospital –cannot afford to buy adequate equipment and supplies. However, the other barriers could be due to poor management services of the hospital.

Similar to our study, a study conducted in South Africa reported that even though majority of the nurses had access to adequate supplies; most of them strongly affirmed that they needed upgrades. This position was premised on the fact that using foam toothettes/gauze with mouth wash are not very effective. Hence, it has been suggested that supplying patients with a toothbrush on admission could help to prevent complications associated with poor oral care. In addition, the same study noted that for many of the nurses the only source of oral care training was during their basic training. This could be challenging, as oral care for an intubated and non-intubated patient requires a different knowledge sets and skills [8].

Parallel to our study, a systematic review conducted in Iran identified several factors that hindered nurses from carrying out oral care. These factors included nurses’ time constraints, lack of adequate staff, heavy workload, poor supervision, poor teamwork, ineffective training and lack of standard protocols [10]. In contrast, a study in Sudan noted that the main barrier included uncooperative behavior of patient followed by inadequate staff, fear of tube displacement in critical care unit, unpleasant task and lack of knowledge [11]. These findings were corroborated in a subsequent study conducted in India in which uncooperative nature of patients was reported as the main barrier tailed by lack of time [13].

According to the focus group participants and key informants, inadequate knowledge of the nurses and their superiors were the main barriers to proper oral care practice. The only source of training for oral care was during their basic nursing skills practice. Lack of knowledge in proper oral assessment, proper techniques and oral hygiene products were the major lags of knowledge among nurses. Inadequate knowledge of nurses in oral care can lead to lack of identification of early signs of oral cavity infection due to poor oral hygiene. This finding is in agreement with the study done in South Africa and Sudan [8, 11].

Moreover, lack of in-service training was mentioned as a barrier to proper oral care practice. Even though, regular in-service training enriches nurses’ knowledge and skill, they had never been given in-service training and never raised as a topic of interest in the wards. This implies that oral care had been put aside and ignored by both the staff and supervisors. Therefore, nurses should have an updated knowledge on assessment skills to deliver oral care services properly, and there must be training and evaluation modalities to enhance nurses’ information on oral care nursing practice.

Another barrier to oral care has been shortage of adequate oral care equipment and supplies. This finding is consistent with the studies done in South Africa, Iran and Sudan [8, 10, 11]. In our study nurses were performing oral care by swabbing the mouth using gauze, washing with normal saline or tap water and occasionally using lemon juice. However, normal saline may cause dryness of the oral mucosa and tap water may be contaminated with nosocomial bacteria which are inefficient and harmful methods of oral care. On the other hand, lemon juice may deplete the salivary reflex mechanism causing xerostomia and decalcification of tooth [16]. Hence, the nurses in our study have been using ineffective oral care methods which is inconsistent with the current CDCs oral health recommendations [5]. Therefore, availability of effective products and supplies are crucial to facilitate provision of oral care.

The absence of a guideline was also stated as a barrier to oral care practice by the participants. Even though, a guideline or standard protocol does not guarantee good oral care practice, it enables for a uniform care of all patients. In the current study, the staff nurse participants had stated that they did not have oral care guidelines in their wards while the key informants and head nurses denied that they were not available. Moreover, the guideline was not updated and it also did not include oral health and systemic diseases, oral care for intubated patients, list of equipment and supplies necessary for effective oral care for hospitalized patients. Our finding is concurrent with the studies done in South Africa, Iran and Sudan [8, 10, 11]. Hence, it can be suggested that there is a gap of supervision and auditing of the necessary materials for oral care practice.

Among the barriers of oral care practice, shortage of staff nurses was mentioned by the participants. Shortage of nurses is a structural variable that puts pressure on nurses and leads to feelings of disappointment or discontent, and powerlessness. In addition, scarcity of nurses will lead to increased work load of nurses, consequently nurses will give more priority to other nursing activities. Our finding is consistent to the studies done in Iran and Sudan [10, 11]. Hence, oral care has been given low priority and led to nurses impeding oral care of patients.

The perception of nurses to oral care as well as their lack of initiative were also among the barriers identified by the participants. Oral care has been undervalued by nurses in the hospital. This may be attributed to the fact that nurses had poor knowledge and awareness regarding oral care as well as lack of supervision by their superiors. In addition, lack of educational advancement, motivation and satisfaction of the nurses can affect the perception of the nurses negatively towards oral care practice in the wards. Equally, poor initiative of nurses was a major contributing factor to inadequate oral care for hospitalized patients.

This study also discovered management gaps as a barrier in the oral care provision of patients. The management gaps were in supervision, team work and oral care plan. Poor supervision concerning oral care by the heads of each ward as well as the matron office was principally due to the lack of knowledge they had in oral care. Consequently, there would not be a controlling or assessment measure for the nurses since oral care was not part of the daily nursing care plan. Another concern was also lack of team work among the nurses and their superiors which has led to ineffective communication.

## Conclusions

The study findings indicated that standard oral care protocol based practice for hospitalized patients was not satisfactorily performed. Lack of knowledge to deliver effective oral care, and inadequate resources were among the main barriers stated by all participants. In addition, poor initiative, underestimation of oral care by the nurses, poor supervision and management gap were among the barriers to oral care. Hence, the present study concluded that nurses lack adequate knowledge and practice of oral care to hospitalized patients due to individual, organizational and ministry level barriers. The study, therefore, suggests a need for regular in-service training and further education, provision of equipment, standard protocol, strong supervision and equalizing nurse to patient ratio.

## Supplementary information

**Additional file 1.** Questionnaire for obtaining nurses’ barriers to quality oral care practice at a generalized hospital in Asmara, Eritrea.

**Additional file 2.** Focus group discussion and key informant interview guide to assess nurses’ barriers to quality oral care practice at a generalized hospital in Asmara, Eritrea.

## Data Availability

The complete dataset supporting the conclusions of this article is available from the corresponding author and can be accessed upon a reasonable request.

## Abbreviations

ENT: Ear, Nose and Throat
FGD: Focus Group discussion
ICU: Intensive Care Unit
IQR: Interquartile range
KII: Key Informant Interview
MOH: Ministry of Health
NCD’s: Non-Communicable Diseases
SD: Standard Deviation
WHO: World Health Organization
