# Supplementary material for: Do nurses have barriers to quality oral care practice at a generalized hospital care in Asmara, Eritrea? A cross-sectional study
Source: BMC Oral Health. 2020 May 20;20:149. doi: 10.1186/s12903-020-01138-y (PMC7240980; doi:10.1186/s12903-020-01138-y)
Supplement: Supplementary file 1 — Additional file 1. Questionnaire for obtaining nurses’ barriers to quality oral care practice at a generalized hospital in Asmara, Eritrea. [file 12903_2020_1138_MOESM1_ESM.docx]

**Additional file 1**

**Questionnaire**

**Do nurses have barriers to quality oral care practice at a generalized hospital care in Asmara, Eritrea? A cross-sectional study**

| ALL INFORMATION COLLECTED IS CONFIDENTIAL AND IS ONLY FOR STATISTICAL USE | | | | |  |
| --- | --- | --- | --- | --- | --- |
| **IDENTIFICATION** | | | | |  |
| Code ……………………………………………………………………………………………………………………………….  Age…………………………………………………………………………………………………………………………………  Sex………………………………………………………………………………….……………………………………………... | | | |  |  |
| **INTERVIEW** | | | | | |
| DATE OF INTERVIEW _____/____/____/  DD MM YEAR  RESULT OF INTERVIEW  * RESULT CODES  1=COMPLETED 2= PARTIALLY COMPLETED 3= REFUSED 4=RESPONDENT NOT FOUND  5=OTHERS (SPECIFY)____________________ | | | | | |
| INTERVIEWER  NAME_________________  DATE _____/_______/________  DD MM YEAR | | | SUPERVISOR  NAME______________________  DATE _____/_______/________  DD MM YEAR | COMPUTER OPERATOR  NAME_________________  DATE _____/_______/________  DD MM YEAR | |

**INTRODUCTION AND CONSENT**

| ***READ TO PARTICIPANT:***  You have been selected to be part of a study entitled “Do nurses have barriers to quality oral care practice at a generalized hospital care in Asmara, Eritrea? A cross-sectional study, and this is why we would like to interview you. This study is conducted by Zewdi Amanuel Dagnew, Isayas Afewerki Abraham, Ghirmay Ghebreigziabher Beraki, Sibyl Mittler, Oliver Okoth Achila, and Eyasu H. Tesfamariam.  The interview will take approximately 10 minutes. You will be asked some questions about your work as a background, including qualification and experiences at this hospital and other facilities where you work. The information you provide will be used only to understand about the barriers to quality oral care practice.  The information you provide is totally confidential and will not be disclosed to anyone. It will be used only for research purposes and a code will be used to connect your answers with the facility without identifying you. Your participation is voluntary. If you have any questions about this study you can ask as you wish.  Are you willing to participate in this survey?  PARTICIPANT AGREE TO BE INTERVIEWED PARTICIPANT DOES NOT AGREE TO BE INTERVIEWED  Signature:__________________________ END |
| --- |

**Section 1:** Questionnaire on demographic characteristics

| **No** | **Question** | **Coding** | **Skip** |
| --- | --- | --- | --- |
| 101 | Age | ________ |  |
| 102 | Sex | 1. Male 2. Female |  |
| 103 | What is your highest level of education? | 1. Associate nurse 2. Diploma nurse |  |
| 104 | The ward within which you are working | 1. Medical Ward 2. Surgical Ward 3. Emergency Ward 4. ICU ward 5. Recovery Ward |  |
| 105 | What is your work experience in the hospital? | 1. <1year 2. 1-5year 3. 6-10year 4. >10year |  |

**Section 2:** Questionnaire on barriers to oral care practice

| **No** | **Question** | **Coding** | **Skip** |
| --- | --- | --- | --- |
| 201 | Are there any barriers that hinder you from performing oral care? | 1. Yes 2. No | If No →End |
| 202 | What factors do you think hinder or prevent you from doing oral care? | 1. Lack of oral care equipment 2. Time constraint 3. Shortage of nurses 4. Lack of knowledge 5. Absence of guideline 6. No on the job training 7. Poor supervision 8. High workload 9. It’s not our priority 10. Not enthusiastic |  |
