# Supplementary material for: Do nurses have barriers to quality oral care practice at a generalized hospital care in Asmara, Eritrea? A cross-sectional study
Source: BMC Oral Health. 2020 May 20;20:149. doi: 10.1186/s12903-020-01138-y (PMC7240980; doi:10.1186/s12903-020-01138-y)
Supplement: Supplementary file 2 — Additional file 2. Focus group discussion and key informant interview guide to assess nurses’ barriers to quality oral care practice at a generalized hospital in Asmara, Eritrea. [file 12903_2020_1138_MOESM2_ESM.docx]

**Additional file 2**

**Consent to participate in Focus Group Discussion (FGD) and Key Informant Interview (KII)**

You are selected to participate in a FGD/KII by the researchers. The purpose of this study focus on barriers to quality oral care practice at your hospital. The information obtained from the FGD and KII will be used to identify the barriers that impede nurse’s to perform quality oral care and to recommend nurses training institutions to improve their curriculum. This study will also recommend the Ministry of Health to provide the hospital with standard protocol/guideline and adequate equipment to perform quality oral care.

You can choose whether to participate/withdraw from the FGD/KII. Even though the FGD and KII will be tape recorded, your responses will remain anonymous and no names will be mentioned in the report. There are no right or wrong answers to the questions. We want to hear many different viewpoints and would like to hear from everyone.

In the FGD, in respect for each other, only one individual speaks at a time in the group and responses made by all participants will be kept confidential. We hope you can be honest even when your responses may not be in agreement with the rest of the participants.

I understand this information and agree to participate fully under the conditions stated above:

**Signature: ________________________________ Date: ___________________**

**Demographic characteristics of FGD and KII**

| Date:________________________________  Time:________________________________  Place:_________________________________  Ward:_________________________________ | |
| --- | --- |
| **Age** |  |
| **Sex** |  |
| **Qualification** | **MSN BSN 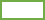 DN 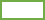 AN 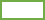** |
| **Position** |  |
| **Years of experience** |  |

**Participants**

- Matron
- Assistant Matron
- Supervisors
- Head nurses
- Staff nurses

**Ground Rules**

- Introduce yourself
- FGD (90mins) and KII (60mins)
- Respect opinion of others -FGD
- Stay with the group and please don’t have side conversations -FGD
- Turn off cell phones if possible

**Questions**

1. How do you see oral care practice in the hospital?
2. Is oral care given routinely in your respective ward? (If Yes, why?)
3. Why do you think is oral care not routine in the hospital?
4. Do you have a guideline that outlines standard oral care protocol in your set up?
   1. **If Yes:**

- Is it applicable?
- Is it regularly updated?
- Is it useful?
- By whom is provided?
  1. **If No, why?** (What is the problem, its priority, lack of awareness on its importance?)

1. Is on the job training been given in your hospital regarding oral care?
   1. **If No, why?** (Have you ever asked for training, source of the problem, how does it affect the quality of oral care practice?)
   2. **If Yes**: (When, was it regularly updated, by whom?)
2. Do you think there are barriers that imped nurses to give oral care to patients?
   1. Lack of oral care equipment
   2. Time constraint
   3. Shortage of nurses
   4. Inadequate knowledge
   5. Absence of guideline
   6. Haven’t been given training
   7. Poor supervision
   8. Too much work to do
   9. It’s not our priority
   10. Not enthusiastic
   11. Others______________

(How could it be solved? Why it is happening? How can this influence the patient’s outcome?)

1. What needs do you want to address regarding these issues? Probe: What specific actions, policy or funding priorities would you need to improve nurse’s practice of oral care?
